# An 8‐year observational study of the death places of patients with COPD in Turkey

**DOI:** 10.1111/ggi.14725

**Published:** 2023-11-03

**Authors:** Aytekin İdikut, Maide Gözde İnam, Damla Karadeniz Güven, Serdar Ceylan, Oğuz Karcioğlu

**Affiliations:** ^1^ Department of Chest Diseases, Faculty of Medicine Hacettepe University Ankara Turkey; ^2^ Department of Internal Medicine, Division of Geriatrics, Faculty of Medicine Hacettepe University Ankara Turkey

**Keywords:** decedent, end of life, lung diseases, pandemic, place of death

## Abstract

**Aim:**

The place of death is one of the indicators of the quality of end‐of‐life care, which has become an essential public health issue with the aging of the population and the increase in life expectancy. There is a lack of data regarding the location of deaths caused by chronic obstructive pulmonary disease (COPD), the third‐leading cause of mortality worldwide. This retrospective, cross‐sectional study aimed to investigate the places of death of patients with COPD in Turkey and their trends over the years.

**Methods:**

The study included patients who had a COPD International Classification of Diseases code in the hospital information system and were provided a medication report for this disease in a university hospital's chest diseases outpatient clinic between January 1, 2014, and December 31, 2021. The place and date of death were obtained from the death notification system and recorded as an in‐hospital or out‐of‐hospital death.

**Results:**

A total of 1402 (77.3%) patients died in the hospital and 412 (22.7%) died outside the hospital, and when comparing the pandemic period and before, no significant difference was observed between the places of death. Sixty‐three (49.6%) of 127 patients over the age of 90 years died outside the hospital, and a significant relationship was observed between advanced age and out‐of‐hospital mortality (*P* < 0.005).

**Conclusion:**

According to our findings, a substantial number of patients with COPD in Turkey die in hospitals. The insufficiency of nursing homes and lack of hospice care cause more hospital deaths. Our data are expected to guide the development of end‐of‐life care policies for patients with COPD in our country. **Geriatr Gerontol Int 2023; 23: 938–944**.

## Graphical abstract

Age was associated with an increase in the home mortality rate (*P* < 0.005). A higher in‐hospital death rate is associated with a history of cardiovascular diseases and malignancy (*P* = 0.01 and *P* = 0.05, respectively). Place of death, which is an important indicator of end‐of‐life care, may vary depending on many factors such as the preferences of patients and their families and the effectiveness of health care systems and needs to be examined comprehensively.

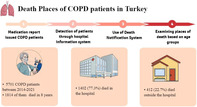

## Introduction

Chronic obstructive pulmonary disease (COPD) is the third‐leading cause of death and the seventh leading cause of disability‐adjusted life years (DALYs). It imposes a significant economic burden on countries.^1^ It caused 3.23 million deaths in 2019,^2^ and it is estimated that the prevalence and mortality rate of COPD will increase with the world's aging. Aging is a major risk factor for COPD due to age‐related decline in respiratory function and prolonged exposure to harmful gases over time.^3^ In addition, increasing life expectancy makes COPD a problem for aging individuals and society. In recent years, the number of people living with COPD and how and where they lived have become more important than how many people died due to COPD. End‐of‐life care (ELC) does not reduce the care of patients to symptom control but also focuses on improving the physical and psychological well‐being of both patients and their relatives, and even around where and how they will die.^4^

The place of death is an important aspect of ELC because where patients die determines how they will die. Although the interaction of many factors, such as causes of death, palliative care programs, social conditions, home care, and nursing facilities, determines where people die, planning the place of death allows for more qualified end‐of‐life care for patients and their caregivers. Knowing where the patient will live at the end of life and where to die is essential for planning ELC for families and governments.

End‐of‐life care planning and determination of the place of death are employed more efficiently in patients with malignancies.^5^ The gradual deterioration of malignant diseases and the realization that they will result in death may lead patients with cancer to accept these ideas more readily. In recent years, an increase in out‐of‐hospital deaths has been observed in developed countries using home‐based ELC programs.^6^, ^7^ Although COPD causes more deaths and DALYs than most malignant diseases, our information about ELC and the place of death is mainly derived from studies on patients with malignancies.^8^

There is a lack of data on the places of death for patients with COPD in Turkey. This retrospective, cross‐sectional investigation aimed to examine the places of death of patients with COPD in Turkey and changes in trends over time.

## Methods

### Study design

This was a single‐center, retrospective study. The study included patients who had a COPD International Classification of Diseases (ICD) code in the hospital information system and were provided a medication report for this disease in a university hospital's chest diseases outpatient clinic between January 1, 2014, and December 31, 2021. They were selected among adult patients whose COPD ICD code was entered and who applied to the chest diseases clinic as an outpatient for any reason, and for whom a medication report was issued for this diagnosis between 2014 and 2021. The location and date of death were obtained from the death notification system and recorded as an in‐hospital or out‐of‐hospital death. On January 1, 2013, Turkey implemented the death notification system for burial procedures. The Death Notification System is a web application that allows data exchange between relevant Ministry of Health units, the General Directorate of Population and Citizenship Affairs, and the Turkish Statistical Institute to compile death statistics in a more complete, faster, and higher‐quality manner. It is expandable and can be managed in a single database and corporate hierarchical structure. The government required registration in this system; thus, data inclusion began in 2013. Information from previous years is insufficient and untrustworthy for assessing accurately.^9^ As a result, deaths occurring on or after January 1, 2014, were examined because there were few reliable data before that date. A hospital information system was used to record demographic and clinical data.

### Ethics approval

The study protocol adhered to the principles of the Declaration of Helsinki. The university's local ethics committee approved the study protocol (GO: 22/221, April 17, 2022).

### Statistical analysis

IBM SPSS software version 24.0 was used for statistical analysis. Descriptive statistics were used. Pearson's chi‐square test was used to investigate the relationship between the location of death and the categorized variables. Logistic regression analysis was performed to investigate factors that independently influenced the place of death. We used a *P*‐value <0.05 to determine statistical significance.

## Results

**Figure 1:**
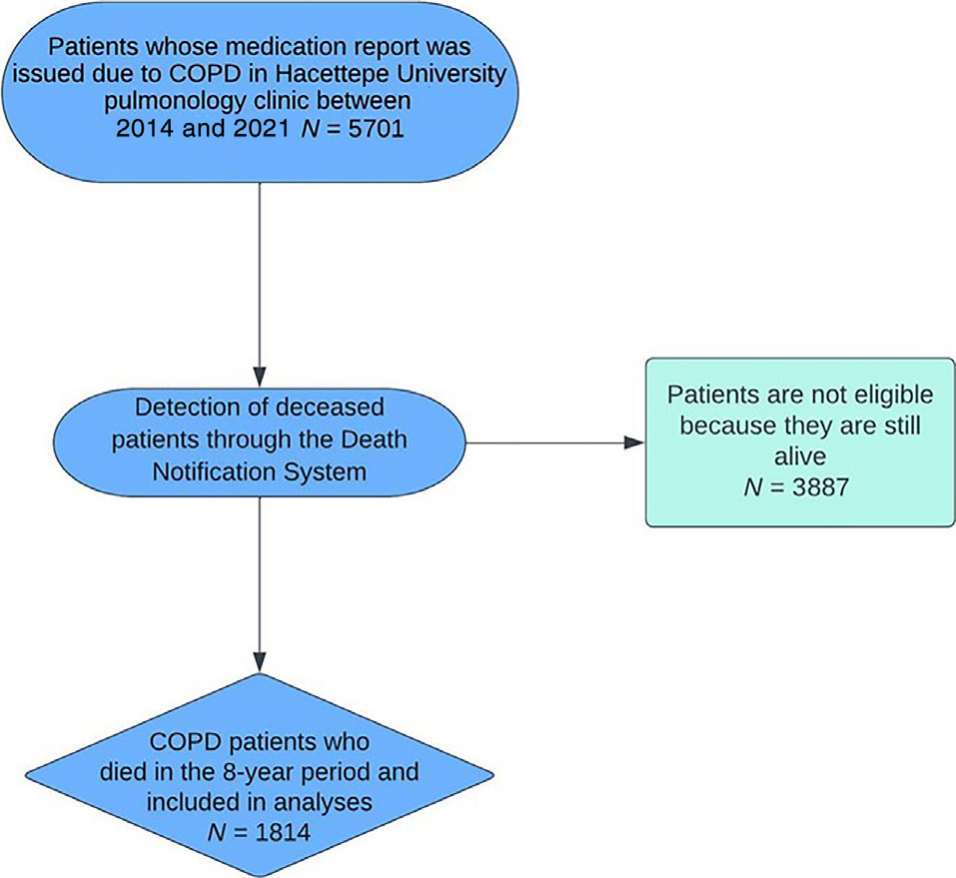
Selection of patients included in the study. COPD, chronic obstructive pulmonary disease.

**Figure 2:**
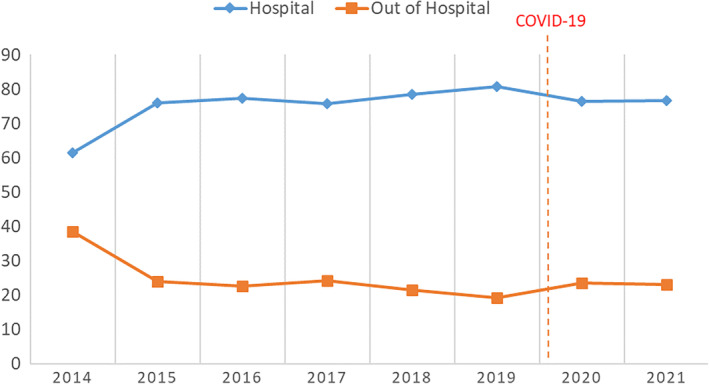
Changes in death places by years.

Screening of 5701 people diagnosed with COPD between 2014 and 2021 revealed a total of 1814 deaths (Figure 1), of which 1402 (77.3%) occurred in the hospital and 412 (22.7%) occurred out of the hospital, and 38.5% (*n* = 698) of the deceased were women. The in‐hospital mortality rates were 78.1% for women and 76.8% for men. Considering the sex distribution, no significant difference was observed in the places of death (*P* < 0.52 for women) (Table 1). The median age was 75.0 (interquartile range [IQR], 69.0–82.0), 84.0 (IQR, 78.0–90.0), and 77.0 (IQR, 70.0–84.0) for those who died in the hospital, out of the hospital, and overall, respectively (Tables 1 and 2). The out‐of‐hospital mortality rate increased with age (*P* < 0.001). It was discovered that patients over the age 90 years died out of the hospital at a higher rate than other age groups (*P* = 0.025). There was no significant change in the place of death over the years (2014–2021) (*P* = 0.47) (Table 2, Figure 2).

**Table 1:** Demographic and clinical features^†^

	Female (*N* = 698, 38.5%) (*N*, %)	Male (*N* = 1116, 61.5%) (*N*, %)	Total (*N* = 1814) (*N*, %)
Age (median)	78.0 (IQR, 71.0–85.0)	77.0 (IQR, 70.0–84.0)	77.0 (IQR, 70.0–84.0)
Place of death (hospital)	545 (78.1)	857 (76.8)	1402 (77.3)
Dementia	83 (11.9)	72 (6.5)	155 (8.5)
Heart failure	240 (34.4)	293 (26.3)	533 (29.4)
Cerebrovascular disease	113 (16.2)	150 (13.4)	263 (14.5)
Cancer	232 (33.2)	513 (46.0)	745 (41.1)
Chronic kidney disease	118 (16.9)	179 (16.0)	297 (16.4)
Age group (10‐year)			
40–49	10 (1.4)	10 (0.9)	20 (1.1)
50–59	30 (4.3)	44 (3.9)	74 (4.1)
60–69	108 (15.5)	208 (18.6)	316 (17.4)
70–79	246 (35.2)	423 (37.9)	669 (36.9)
80–89	250 (35.8)	358 (32.1)	608 (33.5)
≥90	54 (7.7)	73 (6.5)	127 (7.0)
Death years			
2014	11 (1.6)	15 (1.3)	26 (1.4)
2015	64 (9.2)	82 (7.3)	146 (8.0)
2016	77 (11.0)	108 (9.7)	185 (10.2)
2017	86 (12.3)	144 (12.9)	230 (12.7)
2018	114 (16.3)	170 (15.2)	284 (15.7)
2019	107 (15.3)	194 (17.4)	301 (16.6)
2020	126 (18.1)	214 (19.2)	340 (18.7)
2021	113 (16.2)	189 (16.9)	302 (16.6)
Before pandemic	483 (69,2)	754 (67,6)	1237 (68.2)
After pandemic	215 (30.8)	362 (32.4)	577 (31.8)

IQR, interquartile range.

^†^ Percentages are percent of the column.

**Table 2:** Demographic and clinical features by place of death

	Before pandemic (*N* =1237, 68.2%)		*P*	During pandemic (*N* = 577, 31.8%)		*P*	Total (*N* =1814)		*P*
	Hospital (*N* = 962, 77.8%) (*N*, %)	Out of hospital (*N* = 275, 22.2%) (*N*, %)		Hospital (*N* = 440, 76.3%) (*N*, %)	Out of hospital (*N* = 137, 23.7%) (*N*, %)		Hospital (*N* = 1402, 77.3%) (*N*, %)	Out of hospital (*N*: 412, 22.7%) (*N*, %)	
Age (median)	76.0 (IQR, 69.0–82.0)	84.0 (IQR, 78.0–88.0)	<0.001	75.0 (IQR, 68.0–81.0)	82.0 (IQR, 76.0–86.5)	<0.001	75.0 (IQR, 69.0–82.0)	84.0 (IQR, 78.0–90.0)	<0.001
Sex (Female)^†^			0.96			0.31			0.52
	376 (39.1)	107 (38.9)		169 (38.4)	46 (33.6)		545 (38.9)	153 (37.1)	
Dementia^†^			0.07			0.90			0.12
	75 (7.8)	31 (11.3)		37 (8.4)	12 (8.8)		112 (8.0)	43 (10.4)	
Heart failure^†^			0.26			0.17			0.08
	293 (30.5)	74 (26.9)		133 (30.2)	33 (24.1)		426 (30.4)	107 (26.0)	
Cerebrovascular disease^†^			0.93			0.91			0.97
	124 (12.9)	36 (13.1)		79 (18.0)	24 (17.5)		203 (14.5)	60 (14.6)	
Cancer^†^			0.19			0.14			0.05
	424 (44.1)	109 (39.6)		169 (38.4)	43 (31.4)		593 (42.3)	152 (36.9)	
Chronic kidney disease^†^			0.09			0.05			0.01
	167 (17.4)	36 (13.1)		79 (18.0)	15 (10.9)		246 (17.5)	51 (12.4)	
Age group (10‐year)*			<0.001			<0.001			<0.001
40–49	14 (100.0)	0 (0.0)		5 (83.3)	1 (16.7)		19 (95)	1 (5.0)	
50–59	47 (94.0)	3 (6.0)		23 (95.8)	1 (4.2)		70 (94.6)	4 (5.4)	
60–69	195 (94.7)	11 (5.3)		104 (94.5)	6 (5.5)		299 (94.6)	17 (5.4)	
70–79	380 (83.7)	74 (16.3)		171 (79.5)	44 (20.5)		551 (82.4)	118 (17.6)	
80–89	276 (66.0)	142 (34.0)		123 (64.7)	67 (35.3)		399 (65.6)	209 (34.4)	
≥90	50 (52.6)	45 (47.4)		14 (43.8)	18 (56.3)		64 (50.4)	63 (49.6)	

IQR, interquartile range.

^†^ Percentages are percent of the column.

* Percentages are percent of the row.

The most common comorbidities were cancer (41.1%, *n* = 745), congestive heart failure (CHF; 29.4%, *n* = 533), chronic kidney disease (CKD; 16.4%, *n* = 297), and cerebrovascular disease (14.5%, n = 263). Significantly higher in‐hospital mortality was observed among patients with COPD with CKD and cancer (*P* = 0.01 and *P* = 0.05, respectively). CHF, dementia, Parkinson disease, and cerebrovascular diseases were not shown to cause a significant change in the place of death (Tables 1 and 2).

By comparing before and after March 11, 2020, the date of the first COVID‐19 case in Turkey, we investigated whether the pandemic affected the places of death of patients with COPD. The median age of in‐hospital death was 76.0 (IQR, 69.0–82.0) before the pandemic and 75.0 (IQR, 68.0–81.0) during the pandemic. The in‐hospital death rate was 77.8% before and 76.3% during the pandemic. No significant differences were found in the place of death (*P* = 0.47; Table 2). However, while there were 1237 (68.2%) deaths between January 2014 and March 2020 (200.8/year), there were 577 (31.8%) deaths in the 2 years between March 2020 and December 2021 (315.3/year), indicating that mortality increased during the pandemic regardless of the place of death (Tables 2 and 3).

**Table 3:** Place of death in age groups by death years^†^

Age groups	Place of death	Years									*P*
		2014 (*N*, %)	2015 (*N*, %)	2016 (*N*, %)	2017 (*N*, %)	2018 (*N*, %)	2019 (*N*, %)	2020 (*N*, %)	2021 (*N*, %)	Total (*N*, %)	
40–49											0.87
	Hospital	0 (0.0)	1 (100)	2 (100)	1 (100)	4 (100)	4 (100)	2 (100)	5 (83.3)	19 (95.0)	
	Out of hospital	0 (0.0)	0 (0.0)	0 (0.0)	0 (0.0)	0 (0.0)	0 (0.0)	0 (0.0)	1 (16.7)	1 (5.0)	
50–59											0.86
	Hospital	3 (100)	4 (100)	7 (100)	8 (100)	8 (100)	16 (88.9)	14 (93.3)	10 (90.9)	70 (94.6)	
	Out of hospital	0 (0.0)	0 (0.0)	0 (0.0)	0 (0.0)	0 (0.0)	2 (11.1)	1 (6.7)	1 (9.1)	4 (5.4)	
60–69											0.37
	Hospital	0 (0.0)	25 (96.2)	29 (100)	34 (87.2)	45 (93.8)	54 (96.4)	54 (94.7)	58 (95.1)	299 (94.6)	
	Out of hospital	0 (0.0)	1 (3.8)	0 (0.0)	5 (12.8)	3 (6.2)	2 (3.6)	3 (5.3)	3 (4.9)	17 (5.4)	
70–79											0.43
	Hospital	8 (66.7)	36 (85.7)	62 (83.8)	68 (80.0)	93 (83,0)	91 (88,3)	109 (82.0)	84 (77,8)	551 (82.4)	
	Out of hospital	4 (33.3)	6 (14.3)	12 (16.2)	17 (20.0)	19 (17.0)	12 (11.7)	24 (18.0)	24 (22.2)	118 (17.6)	
80–89											0.83
	Hospital	4 (50.0)	39 (60.9)	38 (62.3)	51 (68.9)	65 (67.0)	64 (68.8)	70 (62.5)	68 (68.7)	399 (65.6)	
	Out of hospital	4 (50.0)	25 (39.1)	23 (37.7)	23 (31.1)	32 (33.0)	29 (31.2)	42 (37.5)	31 (31.3)	209 (34.4)	
≥90											0.93
	Hospital	1 (33.3)	6 (67.7)	5 (41.7)	12 (52.2)	8 (53.3)	14 (51.9)	11 (52.4)	7 (41.2)	64 (50.4)	
	Out of hospital	2 (66.7)	3 (33.3)	7 (58.3)	11 (47.8)	7 (46.7)	13 (48.1)	10 (47.6)	10 (58.8)	63 (49.6)	

IQR, interquartile range.

^†^ Percentages are percent of the row.

Multivariate regression analysis consisting of age, sex, and comorbidities (dementia, heart failure, cerebrovascular illness, chronic renal failure, and cancer) for factors affecting hospital death revealed that younger age increased the likelihood of hospital death (for age odds ratio, 1.11 [95% confidence interval, 1.10–1.13]; *P* < 0.001).

## Discussion

In this retrospective, cross‐sectional study, we found that most patients with COPD died in the hospital during the past 8 years. The ratio of out‐of‐hospital mortality increased with patient age but not over time. A history of CKD and cancer is related to increased in‐hospital mortality. Although the number of deaths per year increased with COVID‐19, the place of death was similar to the pre‐COVID period.

Aging is a significant risk factor for COPD due to age‐related decline in respiratory function and prolonged exposure to harmful gases over time.^3^ In addition, increasing life expectancy makes COPD a problem for aging individuals and society. In recent years, it has become more important to understand how many people live with COPD and how and where they live and die, rather than how many people die due to COPD. The term “end‐of‐life care” does not limit the care of patients to symptom control alone. Instead, it focuses on enhancing the physical and psychological well‐being of patients and their relatives and determining where and how they will die.^4^ Therefore, the place of death is accepted as an indicator of end‐of‐life quality. A comprehensive review of the place of death preferences found that most people want to die at home.^8^ Similarly, Skorstengaard *et al*. showed that most patients preferred to be cared for and died at home, regardless of whether they had malignant or nonmalignant lung and heart diseases.^10^ This may be related to the worry of being subjected to several hurtful and ineffective interventions during hospitalization.

Most published studies on the place of death are related to malignant diseases.^11^, ^12^ Although it causes more debilitating symptoms, such as breathlessness, anxiety, and fear of suffocating and dying, very few studies have focused on patients with COPD. A study investigating places of death in respiratory diseases in the United States revealed that 34.2% of patients with COPD died in the hospital, 29.1% died at home, and the remainder died at nursing homes, hospices, and other places. It is also stated that hospital deaths decreased. while deaths at home and in hospices increased during the study period (2003–2017).^13^ We found that 77.3% of the patients with COPD died in the hospital during the study period. Although we do not have detailed information about out‐of‐hospital deaths, considering the low number of nursing homes and the lack of hospice care in Turkey, we can assume that “out‐of‐hospital” means “home.” Therefore, the home deaths of patients with COPD are comparable in Turkey (22.7%) and the United States (29.5%). From this perspective, we may claim that the difference is not attributable to the decision to die at home but rather to the differences in options other than home and hospital. Similarly, the proportion of in‐hospital deaths decreased in this study, whereas it did not change in our study, probably due to disparity in opportunities. In another, similar study conducted in Spain, it was observed that while the proportion of deaths at home has remained stable in recent years, deaths in hospitals were transferred to nursing homes.^14^ In addition to the change in patient and doctor preferences, the development of the country's health systems also influences the patient's place of death.

As the population ages, COPD prevalence and comorbidities increase, making COPD a principal component of multimorbidity. Common comorbidities in patients with COPD are cardiovascular diseases^15^ (congestive heart failure, arrhythmias, ischemic heart diseases), hypertension,^16^ lung cancer,^17^ sleep disturbances,^18^ cerebrovascular diseases,^19^ and dementia.^20^ Comorbidities may affect the course of COPD, trigger severe exacerbations, or be the primary cause of death in individuals. Gudmundsson *et al*. demonstrated that extrapulmonary comorbidities were the primary cause of death in 40% of patients with COPD with a history of exacerbation.^21^ The presence of comorbidities also affects the death places of the patients. In our study, the presence of CKD and cancer was associated with in‐hospital mortality. This association may be due to deaths during hospitalization for cancer treatment or CKD complications.

ELC includes symptom control, anxiety management, pulmonary rehabilitation, nutrition, and decisions regarding resuscitation and place of death.^22^ Patients and their caregivers may feel less anxious about the end‐of‐life phase if they receive proper supportive care and know where and how to die. A study investigating preferred places for care and death of patients with malignant and nonmalignant diseases revealed that the majority preferred homes, hospices, and nursing homes as death places regardless of their diagnosis. Less than 10% of patients choose to receive ELC and pass away in the hospital.^10^ Recent studies have shown that this is not just a preference but also a reality. Cross *et al*. reported that COPD deaths at home and hospices increased between 2003 and 2017, while hospital deaths decreased.^13^ Another study from Spain revealed that home deaths decreased slightly, nursing home deaths increased, and hospital deaths remained unchanged during 2009–2017.^14^ In contrast, there was no change over time in the present study. This difference may be due to health system differences and countries' opportunities. In a 14‐country study showing the importance of differences in health systems, the percentage of patients with COPD who died at home ranged from 10.4% to 55.4%, the percentage of those who died in the hospital ranged from 41.8% to 78.9%, and the percentage of those who died in nursing homes ranged from 1.5% to 35.4%, which shows the importance of the differences in health systems.^23^ In Turkey, the lack of hospice care and the inadequacy of nursing homes make hospitals the only alternative to the home for dying; unfortunately, this has not changed in the past 8 years.

People prefer to die out of the hospital, especially at home, as they age, probably because the idea of death begins to settle into their thoughts. People's and their relatives' expectations of health centers may drop as they get older and near the end of their lives.^8^, ^24^ Similarly, a study examining changes in places of death in all age groups in Belgium demonstrated that being aged ≥85 years was associated with out‐of‐hospital mortality, corroborating our findings.^25^ The fact that the children of the oldest old people are also older adults may explain why most of these patients pass away more out of the hospital, possibly in nursing homes or hospices.^13^

Finally, we compared the number of deaths before and during the COVID‐19 pandemic. There was no change in place of death, but deaths per year increased dramatically.

Our study has some limitations. We investigated medical records to rule out misrecorded diagnoses; nonetheless, additional airway disorders for which the COPD ICD code was entered for inhaler therapy administration but did not match the COPD diagnostic criteria may have been reported by mistake. This study was conducted at a university hospital in one of Turkey's largest cities. These data may not be representative of the entire society. The number of deaths at home may be slightly higher in rural areas than in urban areas. Turkey's currently used death notification system records whether the deaths occurred in or outside the hospital. This is why we could not present deaths that occurred in nursing homes, hospices, and other places.

One of the strengths of the present study is that it covers a long period and includes a large number of patients. Recording the patients' comorbidities allowed us to identify factors associated with the place of death in patients with COPD. The period before and during COVID‐19 demonstrated the effect of this devastating disease on COPD deaths.

Current guidelines advocate ELC that not only relieves the symptoms but also improves the quality of life for patients and their caregivers.^22^ However, this comprehensive approach is often overlooked in daily practice and remains limited to symptom control, leaving patients vulnerable and significantly lowering the health‐related quality of life.^26^, ^27^ The lack of knowledge about ELC for COPD, the limited number of nursing homes and hospices, and the inability to predict life expectancy in COPD and it's devastating symptoms, such as dyspnea and suffocation, which drive patients to the hospital, all contribute to a high number of hospital deaths.^23^ While attempts at ELC are increasing in Turkey, there have not been enough official and scientific publications on the place of death. A recent study of geriatric patients showed that 75% of deaths occurred in hospitals. According to this study, people with chronic renal failure died predominantly in hospitals. It was also observed that most patients with heart failure died in hospitals, although this was not statistically significant.^24^ Hospitalization may be high because patients with chronic renal failure may need dialysis and patients with heart failure may need intravenous diuretic or inotropic treatment. In addition, hospital deaths may still be high due to the need for noninvasive mechanical ventilation of patients with COPD and the inadequacy of palliative care centers. To the best of our knowledge, no other research has investigated the places of death of patients with COPD in Turkey.

The place of death is determined by the interaction of many factors, including patients’ and their relatives' preferences, the adequacy of health care systems, and the population's social structure. Although ELC is recommended for patients with COPD, studies on this subject are limited. Our data are expected to drive the development of policies for ELC for patients with COPD in Turkey. Undoubtedly, more comprehensive and prospectively designed studies will guide us.

## Disclosure statement

The author(s) declare no potential conflicts of interest with respect to the research, authorship, and/or publication of this article.

## Data Availability

Research data are not shared.
